# Circ-SFMBT2 drives the malignant phenotypes of esophageal cancer by the miR-107-dependent regulation of SLC1A5

**DOI:** 10.1186/s12935-021-02156-8

**Published:** 2021-09-16

**Authors:** Zhiwei Chang, Yang Fu, Yongxu Jia, Ming Gao, Lijie Song, Weijie Zhang, Ruihua Zhao, Yanru Qin

**Affiliations:** Department of Oncology, The First Affiliated Hospital of Zhengzhou University, No.1 Jianshe East Road, Erqi District, Zhengzhou, Henan 450052 People’s Republic of China

**Keywords:** Circ-SFMBT2, Esophageal cancer, miR-107, SLC1A5

## Abstract

**Background:**

Increasing studies focused on the regulatory roles of circular RNAs (circRNAs) in diverse cancers. This study was to evaluate the function and mechanism of circRNA Scm-like with four malignant brain tumor domains 2 (circ-SFMBT2) in esophageal cancer (EC).

**Methods:**

The circ-SFMBT2, microRNA-107 (miR-107) and solute-linked carrier family A1 member 5 (SLC1A5) levels were analyzed by quantitative real-time polymerase chain reaction (qRT-PCR). Cell proliferation was evaluated by 3-(4, 5-dimethylthiazol-2-y1)-2, 5-diphenyl tetrazolium bromide (MTT) assay, colony formation assay and EdU assay. Cell apoptosis and invasion were detected by flow cytometry and transwell assay. Glutamine metabolism was assessed by the corresponding kits for glutamine consumption, α-ketoglutarate production and glutamate production. Western blot was used for protein quantification. The binding analysis was performed using dual-luciferase reporter assay, RNA immunoprecipitation (RIP) and pull-down assays. The functional research of circ-SFMBT2 in vivo was performed by xenograft tumor assay. Exosomes were identified by morphological observation and protein detection.

**Results:**

Circ-SFMBT2 was overexpressed in EC samples and cells. Circ-SFMBT2 downregulation inhibited EC cell proliferation, invasion and glutamine metabolism. Circ-SFMBT2 targeted miR-107 and the regulation of circ-SFMBT2 was achieved by sponging miR-107. SLC1A5 was a target of miR-107, and it worked as an oncogene in EC cells. MiR-107 retarded the EC progression by downregulating SLC1A5. Circ-SFMBT2 could affect the SLC1A5 expression by targeting miR-107. Circ-SFMBT2 regulated EC progression in vivo by miR-107/SLC1A5 axis. Circ-SFMBT2 was transferred by exosomes in EC cells.

**Conclusion:**

These results suggested that circ-SFMBT2 upregulated the SLC1A5 expression to promote the malignant development of EC by serving as a miR-107 sponge.

**Supplementary Information:**

The online version contains supplementary material available at 10.1186/s12935-021-02156-8.

## Background

Esophageal cancer (EC) has been one of the leading causes of cancer-induced human death, in part due to the lack of early detection and precise treatment [1]. EC has intense aggressiveness and a poor prognosis with the overall 5-year survival rate of 10% [2, 3]. Currently, a variety of non-coding RNAs (ncRNAs) have been dysregulated in cancers and they are expected to be the diagnostic or therapeutic targets in cancer management [4].

Circular RNAs (circRNAs) and microRNAs (miRNAs) are well-known subtypes of ncRNAs involving in cancer progression [5]. CircRNAs are endogenous and covalently closed RNAs with tissue/cell-specific expression patterns in eukaryotes [6]. CircRNAs affect the tumor behavioral phenotypes by acting as the post-transcriptional regulators through the “miRNA sponging” mechanism [7]. Wang et al. have found that circRNA-0008717 enhanced cell proliferation and invasion by sponging miR-203 and upregulating the Slug expression in EC cells [8]. Ma et al. reported that EC progression was impeded by circLAPR4 sponging miR-1323 to increase the level of PTEN [9]. CircRNA Scm-like with four malignant brain tumor domains 2 (circ-SFMBT2) has been shown to promote cell proliferation in gastric cancer by the miR-182-5p/CREB1 axis [10]. In addition, circ-SFMBT2 (hsa_circ_0000211) functioned as a carcinogenic factor in lung adenocarcinoma by miR-622/HIF1-α network [11]. GSE131969 dataset (https://www.ncbi.nlm.nih.gov/geo/geo2r/?acc=GSE131969) showed that circ-SFMBT2 was highly expressed in EC tissues. It is unknown about the biological role and mechanism of circ-SFMBT2 in EC.

MicroRNA-107 (miR-107) facilitated the capacities of cell migration and invasion in gastric cancer by targeting FAT4 [12], and it exerted the anti-tumor effect on colorectal cancer by downregulating the expression of TFR1 [13]. MiR-107 was identified as a tumor repressor in the development of EC by the expression inhibition of Cdc42 [14]. The study by Lin et al. demonstrated that solute-linked carrier family A1 member 5 (SLC1A5) knockdown triggered cell cycle retardation and cell apoptosis in EC [15]. It remains to be explored whether circ-SFMBT2 could be a miR-107 sponge and SLC1A5 was a downstream target of miR-107 in the regulation of EC.

Given the dysregulation of circ-SFMBT2 in EC samples, this study aimed to research the functional role of circ-SFMBT2 in EC. Moreover, we focused on the regulation of circ-SFMBT2 on the expression of SLC1A5 by targeting miR-107. The circ-SFMBT2/miR-107/SLC1A5 signal network firstly disclosed the molecular mechanism of circ-SFMBT2 in EC progression.

## Materials and methods

### Human samples

A total of 39 EC patients have undergone surgical resection at the First Affiliated Hospital of Zhengzhou University. EC tissues (n = 39) and normal paracancerous tissues (n = 39) were collected into the new tubes and saved at − 80 °C. The clinical pathological characteristics of EC patients were exhibited in Table 1. Histopathologic identification was performed by hematoxylin–eosin (HE) staining and Immunohistochemistry (IHC) analysis (anti-Ki67, ab15580, Abcam, Cambridge, UK) [16, 17]. This study has obtained the written informed consent from EC patients and the ratification from the Ethics Committee of the First Affiliated Hospital of Zhengzhou University.

**Table 1:** Relationship between circ-SFMBT2 and clinicopathologic features of EC patients

	Characteristics n = 39	circ-SFMBT2 expression		*P* value^a^
		Low(n = 19)	High(n = 20)	
Gender				0.7512
Female	22	10	12	
Male	17	9	8	
Age (years)				0.7475
≤ 60	15	8	7	
> 60	24	11	13	
TNM grade				0.0104*
I + II	18	13	5	
III + IV	21	6	15	
Lymph node metastasis				0.0095*
Positive	23	7	16	
Negative	16	12	4	
Tumor size				0.0225*
≤ 3 cm	15	11	4	
> 3 cm	24	8	16	

*TNM* tumor-node-metas-tasis

^*^P < 0.05

^a^Chi-square test

### CircRNA microarray data analysis

Total RNA was treated with RNase R (Epicentre Technologies, Madison, WI, USA) to remove linear RNAs. The circRNAs were amplified and transcribed using a random priming method (Arraystar Super RNA Labeling Kit; Arraystar; USA). Subsequently, the labeled circRNAs were hybridized to an Arraystar Human circRNA Array (8 × 15 K, Arraystar). Then, the arrays were scanned with an Agilent G2505C scanner and the array images were analyzed by Agilent Feature Extraction software. Quantile normalization and subsequent data processing were conducted through the R software package. Through the volcano plot filtering, circRNAs with significant differential expression were identified. Hierarchical clustering was performed to visualize the circRNA expression distribution between two groups.

### Cell culture and transfection

Human esophageal epithelial cell line (HEEC) and primary EC cell lines (ECA109, KYSE410, KYSE150, TE1) were purchased from BioVector NTCC Inc. (Beijing, China). These EC cell lines are from the same histological type (squamous cell carcinoma). Roswell Park Memorial Institute-1640 (RPMI-1640; Gibco, Carlsbad, CA, USA) containing 10% fetal bovine serum (Gibco) and 1% antibiotics (Gibco) was used for cell culture in a 5% CO_2_ incubator at 37 °C. Small interfering RNA (siRNA) against circ-SFMBT2 or SLC1A5 (si-circ-SFMBT2, si-SLC1A5), mimic or inhibitor for miR-107 (miR-107, anti-miR-107) and the negative controls (si-NC, si-con, miR-NC, anti-miR-NC) were directly bought from GenePharma (Shanghai, China). The pCD5-ciR, pCD5-ciR-circ-SFMBT2 (circ-SFMBT2), pcDNA and pcDNA-SLC1A5 (SLC1A5) plasmids were provided by GENESEED (Guangzhou, China). KYSE150 and TE1 cells were transfected with RNAs (40 nM siRNA, 40 nM mimic, 20 nM inhibitor) or 2 μg plasmids by Lipofectamine™ 3000 (Invitrogen, Carlsbad, CA, USA).

### The quantitative real-time polymerase chain reaction (qRT-PCR) assay

RNA was purified by TRIzol™ Reagent (Invitrogen) from human samples or cells. RNA SuperScript™ IV First-Strand Synthesis System and SYBR™ Green One-Step qPCR Kit (Invitrogen) were used for the reverse transcription and expression analysis of circ-SFMBT2 and mRNAs. The miRNA quantification was performed by TaqMan Advanced miRNA cDNA Synthesis Kit and TaqMan™ Advanced miRNA Assay (Applied Biosystems, Foster City, CA, USA). The data were expressed as the relative expression level by the 2^−∆∆Ct^ method [18]. Glyceraldehyde-phosphate dehydrogenase (GAPDH) and U6 were the internal controls in this study. The stability of circ-SFMBT2 was analyzed by qRT-PCR after total RNA was treated with RNase R (Epicentre Technologies). In addition, the nuclear or cytoplasmic RNA was isolated by PARIS™ Kit (Invitrogen) and the localization of circ-SFMBT2 was determined using qRT-PCR. All primer sequences were shown as below: circ-SFMBT2, 5’-CTGCCAAATTTCCTCTTCCAA-3’ (Forward, F) and 5’-CAACTGTAATGAGGTCTATAGGGCC-3’ (Reverse, R); SFMBT2, 5’-AGTTAGTGTGATTGAAAATGTTGGA-3’ (F) and 5’-AAGGATAGATTTCTGAAGGTGGGT-3’ (R); miR-107, 5’-GCCGAGAGCAGCATTGTACA-3’ (F) and 5’-CAGTGCAGGGTCCGAGGTAT-3’ (R); SLC1A5, 5’-TGGTACGCCCCTGTGGGCA-3’ (F) and 5’-GTGACCCAGCAGGCAGCACA-3’ (R); GAPDH, 5’-GTCTCCTCTGACTTCAACAGC-3’ (F) and 5’-CCACCCTGTTGCTGTAGCCAA-3’ (R); U6, 5’-CTCGCTTCGGCAGCACA-3’ (F) and 5’-AACGCTTCACGAATTTGCGT-3’ (R).

### 3-(4, 5-dimethylthiazol-2-y1)-2, 5-diphenyl tetrazolium bromide (MTT) assay

KYSE150 and TE1 cells were respectively planted onto the 96-well plates with 1 × 10^4^ cells/well. Cells were cultured at 37 °C for 16 h, and different transfection was performed in monolayer cells. Cell proliferation was analyzed using MTT Cell Proliferation Assay Kit (Solarbio, Beijing, China) as per the user’s guide. The absorbance of 490 nm was examined by a microplate reader (Thermo Fisher Scientific, Waltham, MA, USA) at 24, 48 or 72 h.

### Colony formation assay

KYSE150 and TE1 cells were collected after transfection for 24 h, then the 12-well plates were seeded with 200 cells/well. Cells were cultured for 14 days until the white colonies were observed. The colonies were washed with phosphate buffer solution (PBS; Gibco) and fixated with pre-cooled methanol (Beyotime, Shanghai, China) for 10 min. Whereafter, the colony staining was conducted by crystal violet (Beyotime) for 15 min and the colonies containing at least 50 cells were counted using ImageJ software (NIH, Bethesda, MD, USA).

### EdU assay

Cell proliferation was analyzed by EdU Imaging Detection Kit (KeyGEN, Nanjing, China). 5 × 10^4^ cells were plated into the 96-well plates overnight, and EdU working solution was incubated to the transfected cells. Subsequently, cells were fastened with 4% neutral paraformaldehyde and infiltrated with 0.5% Triton X-100 solution. Then, the each well was added with 100 µL Click-iT EdU reaction buffer and cell nucleus was stained with diamidine phenylindole (DAPI). The imaging analysis was performed on the fluorescence microscope (Olympus, Tokyo, Japan), and the merged cells (EdU + DAPI) were labeled as the EdU-positive cells.

### Flow cytometry

Cell apoptosis was examined using Annexin V-fluorescein isothiocyanate (Annexin V-FITC) Apoptosis Detection Kit (Sigma-Aldrich). Cells were harvested using trypsin (Gibco) without Ethylene Diamine Tetraacetic Acid (EDTA), and 3 × 10^5^ cells were suspended with 500 µL Binding Buffer. The tube was added with 5 µL Annexin V-FITC and 5 µL propidium iodide (PI) solution, then the mixture was incubated in the dark for 10 min. Cell detection was instantly performed by a flow cytometer (Applied Biosystems). The apoptotic cells were defined as the stained cells of Annexin V + /PI- (QLR) and Annexin V + /PI + (QUR).

### Transwell assay

Cell invasive ability was determined in the transwell chamber (Corning Inc., Corning, NY, USA) coated with matrigel (Corning Inc.). 5 × 10^4^ cells in PBS were pipetted into the upper chamber and the lower chamber was added with 500 µL cell culture medium. After cell incubation at 37 °C for 24 h, the non-invaded cells on the upper chamber were removed by a sterile cotton swab. The invaded cells from the upper chamber into the lower chamber were fixed and stained with methanol and crystal violet (Beyotime), followed by cell photographing at 100 × magnification by an inverted fluorescence microscope (Olympus). Cells were counted at three arbitrarily selected fields.

### Glutamine metabolism analysis

Cells were transfected for 48 h and the culture medium was collected. The glutamine consumption was detected using the Glutamine Assay Kit (Biovision, Milpitas, CA, USA) following the manufacturer’s protocols. In addition, alpha-ketoglutarate (α-KG) and glutamate concentrations were respectively measured by Alpha-Ketoglutarate Colorimetric Assay Kit and Glutamate Colorimetric Assay Kit (Biovision).

### Western blot

Western blot was performed according to the previous study [19]. Briefly, 40 μg proteins were loaded on 10% sodium dodecyl sulfate polyacrylamide gel electrophoresis (SDS-PAGE) and transferred onto the polyvinylidene fluoride (PVDF) membranes (Sigma-Aldrich). The membranes were incubated with the primary antibodies against Cyclin D1 (ab16663, 1:1000), matrix metalloproteinase 9 (MMP9; ab76003, 1:1000), CD9 (ab92726, 1:1000), CD63 (ab134045, 1:1000), Calnexin (ab22595, 1:1000), SLC1A5 (ab237704, 1:1000), GAPDH (ab6671, 1:1000) at 4 °C overnight, and the Goat Anti-Rabbit IgG H&L secondary antibody (ab205718, 1:3000) at room temperature for 1 h. All antibodies were purchased from Abcam (Cambridge, MA, USA), and diluted in phosphate buffered saline with 0.1% Tween 20 (PBST). GAPDH acted as a reference gene and the protein level was analyzed using the ImageJ software (NIH).

### Dual-luciferase reporter assay

Starbase (http://starbase.sysu.edu.cn) was used for target prediction for circ-SFMBT2 and miR-107. The original sequences of circ-SFMBT2 and SLC1A5 3’UTR containing the binding sites of miR-107 were considered as the wild-type (WT) sequences. The circ-SFMBT2 and SLC1A5 3’UTR sequences after the mutation of miR-107 binding sites were considered as the mutant (MUT) sequences. Luciferase constructs for circ-SFMBT2 (WT-circ-SFMBT2, MUT-circ-SFMBT2) and SLC1A5 (SLC1A5-3’UTR-WT, SLC1A5-3’UTR-MUT) were obtained by cloning the WT or MUT sequence into the pmirGLO luciferase vector (Promega, Madison, WI, USA). KYSE150 and TE1 cells were respectively co-transfected with each construct and miR-107 or miR-NC. Cells were incubated at 37 °C for 48 h, followed by the luciferase analysis by dual-luciferase reporter assay kit (Promega).

### RNA immunoprecipitation (RIP) assay

The immunoprecipitation assay was performed by Imprint® RNA Immunoprecipitation Kit (Sigma-Aldrich). The anti-Argonaute-2 (anti-Ago2) or anti-immunoglobulin G (anti-IgG) coated Protein A magnetic beads were incubated to 1 × 10^6^ EC cells at 4 °Covernight. The protein was removed by proteinase K, and total RNA was extracted for expression analysis of circ-SFMBT2, miR-107 and SLC1A5.

### RNA pull-down assay

Biotin-coupled miR-107 (bio-miR-107) and negative control (bio-miR-NC) (RIBOBIO) were transfected into KYSE150 and TE1 cells for 48 h. Cells were harvested and incubated with streptavidin magnetic beads (Thermo Fisher Scientific) at 4 °C overnight. After the isolation of total RNA, circ-SFMBT2 and SLC1A5 levels were examined using qRT-PCR.

### Tumor xenograft assay

TE1 cells were transfected with lentiviral vector containing short hairpin RNA (shRNA) of circ-SFMBT2 (sh-circ-SFMBT2) or shRNA negative control (sh-NC) to construct the stable cell line. Six-week-old male BALB/c nude mice (n = 14) were bought from Vital River Laboratory Animal Technology Co., Ltd. (Beijing, China). 2 × 10^6^ TE1 cells of sh-NC or sh-circ-SFMBT2 group were subcutaneously injected into the mice (7 mice/group). One week later, tumor size was measured every 3 days followed by the calculation of tumor volume (length × width^2^ × 0.5). At 22 d, all mice were euthanatized by using the flow rate of CO_2_ to displace the air of container. Tumors were excised from mice, and the weight of each tumor was determined on an electronic scale. Total RNA or protein was isolated from tissues, then the expression analysis (for circ-SFMBT2, miR-107 and SLC1A5) was performed by qRT-PCR or western blot. IHC assay was used for the protein detection of SLC1A5, Ki67 and MMP9 in tumors. The procedures were in accordance with the Management and Use Guidelines of Laboratory Animals of NIH. Also, this assay was authorized by the Animal Ethical Committee of the First Affiliated Hospital of Zhengzhou University.

### Exosome assay

KYSE150 and TE1 cells were cultured in DMEM + FBS medium depleted of extracellular vesicles for 48 h, then cells were collected for exosome extraction by the Exosome Extraction Kit (KeyGen) following the producer’s instruction book. Exosomes were observed under the Transmission electron microscopy (TEM; JEM-1400, JEOL, Akishima, Japan). The protein levels of exosomal markers in exosomes and cell extracts were determined by western blot. To analyze the transfer way of circ-SFMBT2, cells were incubated with 2 μg/mL RNase A (Sigma-Aldrich) or 2 μg/mL RNase A + 0.1% Triton X100 (Sigma-Aldrich) for 20 min. Then, the circ_0006174 expression was detected using qRT-PCR. In addition, KYSE150 and TE1 cells were incubated with exosomal inhibitor GW4869 (Catalog #: 2417; Biovision). Cell supernatants were collected and RNase R treatment was used to remove the linear RNAs in exosomes, followed by the circ-SFMBT2 level analysis through qRT-PCR.

### Statistical analysis

Three independent biological replicates with three paralleled samples were conducted in each experiment. Data were expressed by the mean ± standard deviation (SD), and statistical analysis was carried out by SPSS 22.0 (SPSS Inc., Chicago, IL, USA). Survival curves of patients by Kaplan–Meier plot were analyzed using the log-rank test. The linear relation was analyzed by Pearson’s correlation coefficient. The analysis of difference was performed by Student’s *t*-test and one-way analysis of variance (ANOVA) followed by Tukey’s test. The statistical difference was significant when *P* value was less than 0.05.

## Results

### Circ-SFMBT2 was highly expressed in EC samples and cells

**Fig. 1:**
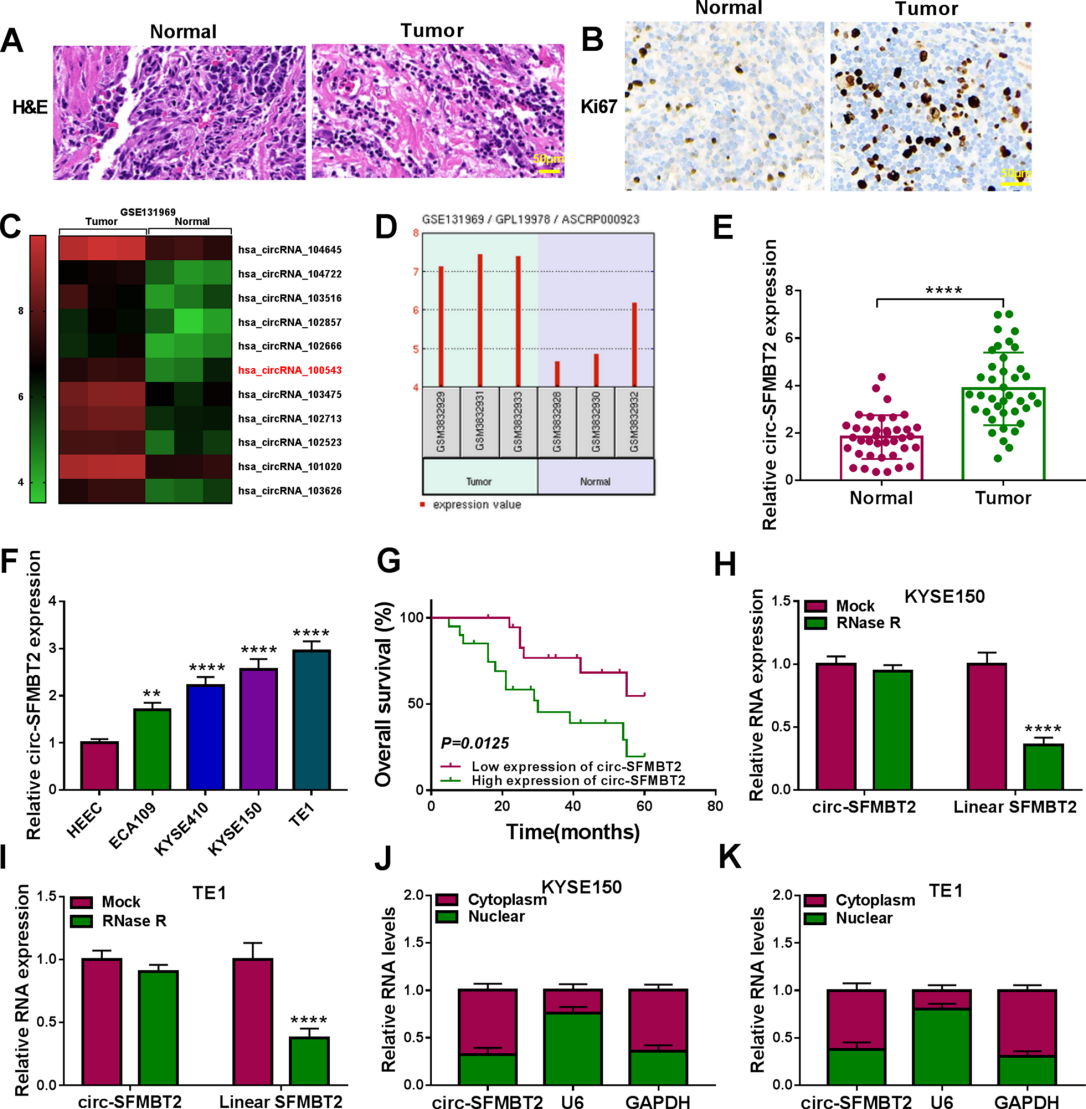
Circ-SFMBT2 was highly expressed in EC samples and cells. **A**, **B** Tumor tissues were identified by HE staining (**A**) and IHC analysis (**B**). **C**, **D** GSE131969 dataset indicated that the expression upregulation of circ-SFMBT2 in EC tissues. **E**, **F** The expression detection for circ-SFMBT2 was conducted by qRT-PCR in normal or EC samples (**E**) and HEEC or EC cells (**F**). **G** The survival analysis was performed by log-rank test. **H**, **I** The stability of circ-SFMBT2 or linear SFMBT2 was analyzed by qRT-PCR after RNase R treatment in total RNA. **J**, **K** The subcellular localization of circ-SFMBT2 by qRT-PCR after the isolation of nuclear and cytoplasmic RNA. **P < 0.01, ****P < 0.0001

HE staining was performed to observe the pathological changes in EC tissues. As shown in Fig. 1A, the esophageal cells of tumor group were in an irregular and disordered arrangement contrasted to the normal group. IHC analysis revealed that Ki67 protein level of tumor group was higher than that of normal group, showing that cell proliferation was promoted in EC samples (Fig. 1B). The dataset GSE131969 (https://www.ncbi.nlm.nih.gov/geo/geo2r/?acc=GSE131969) exhibited that circ-SFMBT2 (hsa_circRNA_100543) was overexpressed in EC tissues (Fig. 1C, D). The qRT-PCR was used for circ-SFMBT2 quantification in our tissues and cells. In comparison to the normal tissues and HEEC cells, the expression of circ-SFMBT2 was signally upregulated in EC tissues (Fig. 1E) and cells (ECA109, KYSE410, KYSE150, TE1) (Fig. 1F). According to the median value, EC patients were divided into high expression group (n = 20) and low expression group (n = 19). The survival curve indicated that the overall survival was decreased in EC patients with high expression of circ-SFMBT2 relative to the patients with low expression of circ-SFMBT2, suggesting that circ-SFMBT2 could be a prognostic target for EC patients (Fig. 1G). Total RNA was treated with RNase R, and circ-SFMBT2 was affirmed to be more stable than linear SFMBT2 (Fig. 1H, I). The abundant circ-SFMBT2 was detected in the cytoplasm of KYSE150 and TE1 cells by contrast to U6 (a nuclear control) and GAPDH (a cytoplasmic control), confirming the cytoplasmic localization of circ-SFMBT2 in EC cells (Fig. 1J, K). Circ-SFMBT2 was differentially expressed in our EC samples and cells.

### Silence of circ-SFMBT2 suppressed proliferation, invasion and glutamine metabolism in EC cells

**Fig. 2:**
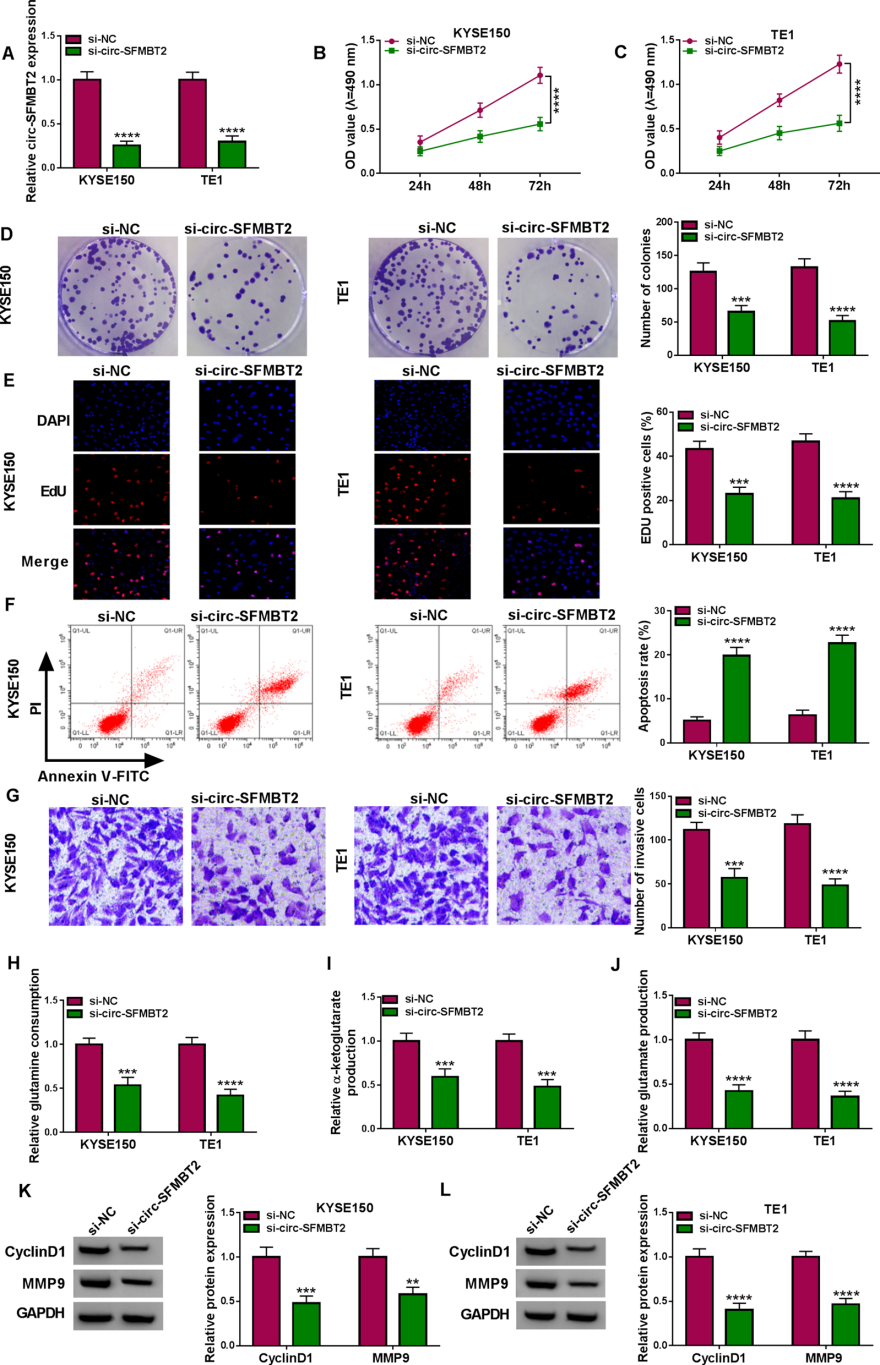
Silence of circ-SFMBT2 suppressed proliferation, invasion and glutamine metabolism in EC cells. Si-NC or si-circ-SFMBT2 was transfected into KYSE150 and TE1 cells. **A** The qRT-PCR was used for the quantification of circ-SFMBT2 expression after transfection for 48 h. **B**, **C** MTT assay was used for the analysis of cell proliferation after transfection for 48 h. **D** Colony formation assay was used for proliferation detection at 24 h post-transfection. **E** EdU assay was used to assess proliferation at 48 h post-transfection. **F** Flow cytometry was used for the examination of cell apoptosis after transfection for 72 h. **G** Transwell assay was applied to evaluate cell invasion after transfection for 24 h. **H**–**J** The glutamine consumption (**H**), α-ketoglutarate production (**I**) and glutamate production (**J**) by the corresponding kits were used for the assessment of glutamine metabolism after cell transfection for 48 h. (K-L) Western blot was used for the protein detection of CyclinD1 and MMP9 after cell transfection for 48 h. **P < 0.01, ***P < 0.001, ****P < 0.0001

To perform the functional exploration of circ-SFMBT2 in EC cells, siRNA was used to interfere the expression of circ-SFMBT2. As presented in Fig. 2A, si-circ-SFMBT2 transfection obviously downregulated the circ-SFMBT2 level relative to si-NC transfection. The effective time of si-circ-SFMBT2 transfection was about 72 h. An inhibitory effect of si-circ-SFMBT2 on cell proliferation was found in KYSE150 and TE1 cells by MTT assay (Fig. 2B, C). Also, cell colonies (Fig. 2D) and EdU-positive cells (Fig. 2E) were reduced in si-circ-SFMBT2 group compared with si-NC group. Flow cytometry and transwell assay demonstrated that knockdown of circ-SFMBT2 evoked the apoptotic acceleration (Fig. 2F) and the invasion inhibition (Fig. 2G). Glutamine is an important nutrient substance for tumor cells since it can convert to glutamate and α-ketoglutarate that is an intermediate in the citric acid cycle [20]. The downregulation of circ-SFMBT2 was shown to repress the glutamine consumption, α-ketoglutarate production and glutamate production in KYSE150 and TE1 cells (Fig. 2H–J). In addition, western blot manifested that the protein levels of CyclinD1 and MMP9 were decreased after the expression suppression of circ-SFMBT2 (Fig. 2K, L). These data suggested that circ-SFMBT2 functioned as a tumorigenic role in EC.

### Circ-SFMBT2 was identified as a miR-107 sponge

**Fig. 3:**
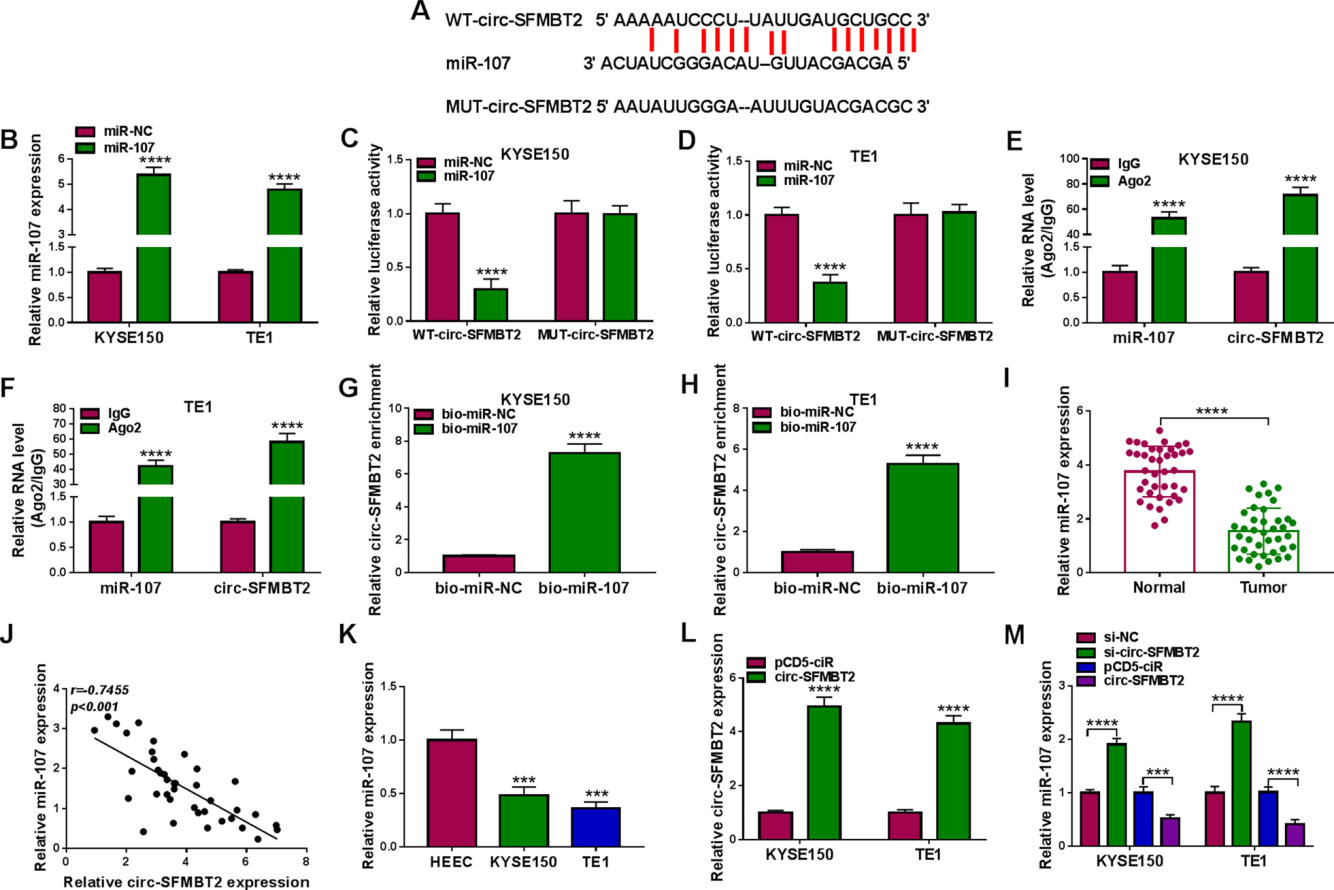
Circ-SFMBT2 was identified as a miR-107 sponge. **A** Starbase predicted the binding sites between miR-107 and circ-SFMBT2. **B** The transfection efficiency of miR-107 was analyzed by qRT-PCR. **C**–**H**) Dual-luciferase reporter assay (**C, D**), RIP assay (**E**, **F**) and RNA pull-down assay (**G**, **H**) were performed to validate the interaction between miR-107 and circ-SFMBT2. **I** The qRT-PCR was performed to examine the miR-107 expression in normal and EC tissues. **J** Pearson’s correlation coefficient was conducted to analyze the linear relation between circ-SFMBT2 and miR-107. **K** The miR-107 level was detected by qRT-PCR in HEEC, KYSE150 and TE1 cells. **L** The overexpression efficiency of circ-SFMBT2 was evaluated using qRT-PCR. **M** The effect of circ-SFMBT2 on the expression of miR-107 was measured using qRT-PCR. ***P < 0.001, ****P < 0.0001

Starbase indicated that circ-SFMBT2 sequence contained the miR-107 binding sites (Fig. 3A). In KYSE150 and TE1 cells, miR-107 level was overexpressed by miR-107 mimic with fivefold changes (Fig. 3B). The miR-107 upregulation could be remained for 72 h after mimic transfection. Dual-luciferase reporter assay was performed to explore whether circ-SFMBT2 could interact with miR-107. As Fig. 3C, D depicted, the overexpression of miR-107 resulted in a repressive effect on the relative luciferase activity of WT-circ-SFMBT2 group instead of MUT-circ-SFMBT2 group. In addition, RIP assay showed that miR-107 and circ-SFMBT2 were enriched by Ago2 protein in KYSE150 and TE1 cells (Fig. 3E, F). Meanwhile, circ-SFMBT2 was pulled down by bio-miR-107 relative to bio-miR-NC group (Fig. 3G-H). The expression analysis for miR-107 exhibited that it was downregulated in EC tissues compared to normal tissues (Fig. 3I). Pearson’s correlation coefficient has indicated a negative relationship (*r* = − 0.7455, *p* < 0.001) between circ-SFMBT2 and miR-107 in EC tissues (Fig. 3J). The low expression of miR-107 was also validated in KYSE150 and TE1 cells by qRT-PCR (Fig. 3K). The transfection of circ-SFMBT2 evidently upregulated the level of circ-SFMBT2 in contrast to the transfection of pCD5-ciR (Fig. 3L). Then the effect of circ-SFMBT2 on the miR-107 expression was determined. The qRT-PCR results revealed that miR-107 level was elevated by knockdown of circ-SFMBT2 but it was inhibited by overexpression of circ-SFMBT2 (Fig. 3M). Thus, circ-SFMBT2 could be a sponge of miR-107 in EC cells.

### The si-circ-SFMBT2-mediated anti-tumor function was reversed by miR-107 inhibitor in EC cells

**Fig. 4:**
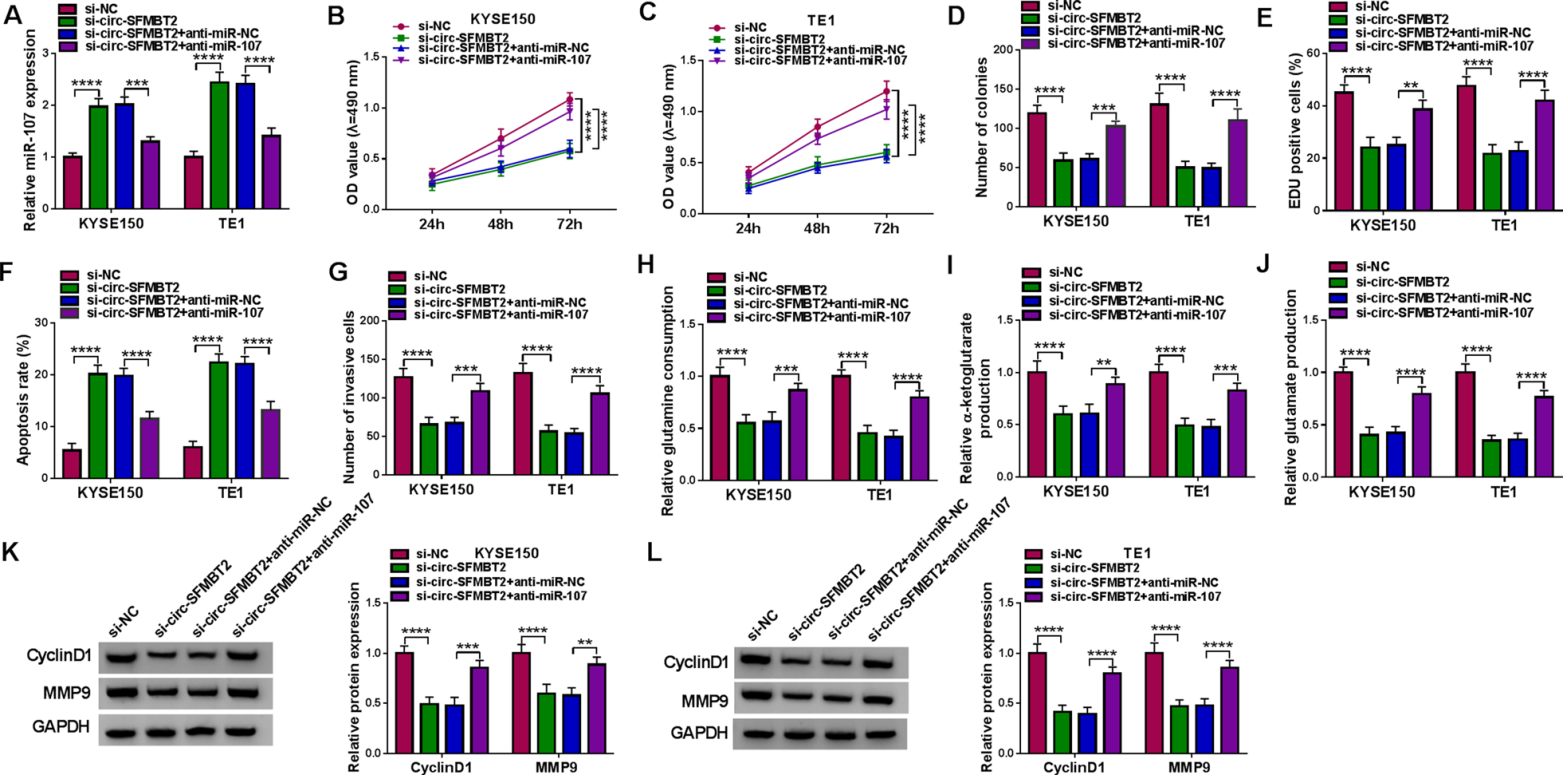
The si-circ-SFMBT2-mediated anti-tumor function was reversed by miR-107 inhibitor in EC cells. KYSE150 and TE1 cells were transfected with si-NC, si-circ-SFMBT2, si-circ-SFMBT2 + anti-miR-NC or si-circ-SFMBT2 + anti-miR-107. **A** The miR-107 expression was assayed using qRT-PCR. **B**–**E** Cell proliferation was examined using MTT assay (**B**–**C)**, colony formation assay (**D**) and EdU assay (**E**). **F**, **G** Cell apoptosis (**F**) and invasion (**G**) were respectively detected using flow cytometry and transwell assay. **H**–**J** Glutamine metabolism was evaluated using the glutamine consumption (**H**), α-ketoglutarate production (**I**) and glutamate production (**J**) by the corresponding kits. **K**, **L** CyclinD1 and MMP9 protein levels were determined using western blot. **P < 0.01, ***P < 0.001, ****P < 0.0001

To prove the association of miR-107 with the function of circ-SFMBT2 in the EC progression, the reverted experiments were performed in EC cells. The miR-107 expression was lower in si-circ-SFMBT2 + anti-miR-107 group than that in si-circ-SFMBT2 + anti-miR-NC group, showing that the efficiency of anti-miR-107 transfection was significant (Fig. 4A). As the results of miR-107 expression downregulation, the inhibition of cell proliferation caused by si-circ-SFMBT2 was abolished (Fig. 4B–E). The pro-apoptotic and anti-invasive effects of si-circ-SFMBT2 were also restored by anti-miR-107 (Fig. 4F, G). Additionally, miR-107 inhibitor alleviated the si-circ-SFMBT2-induced inactivation of glutamine metabolism (Fig. 4H–J) and downregulation of CyclinD1/MMP9 (Fig. 4K, L). It was confirmed that the effect of circ-SFMBT2 on the progression of EC was related to the negative regulation of miR-107.

### SLC1A5 acted as a target for miR-107

**Fig. 5:**
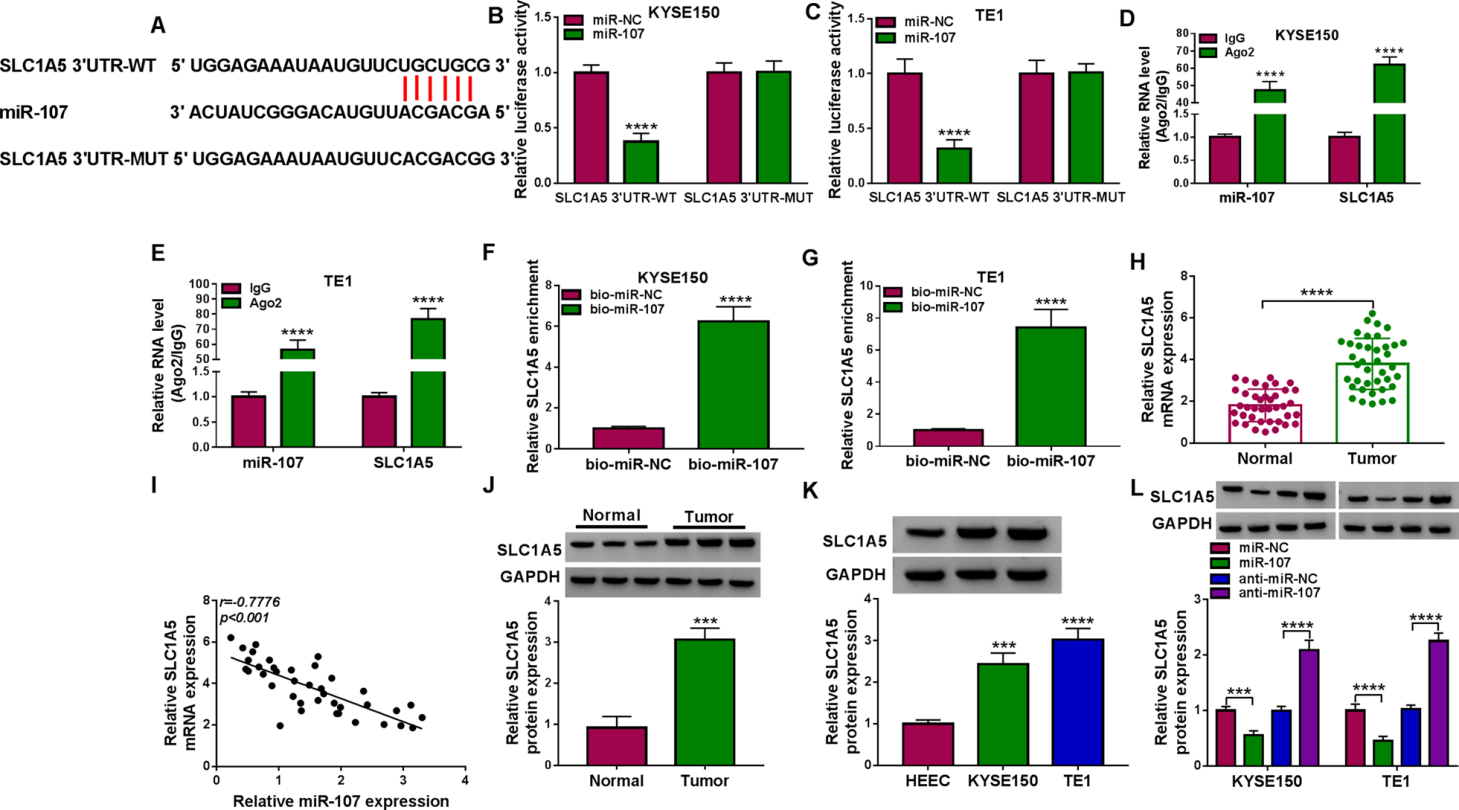
SLC1A5 acted as a target for miR-107. **A** The binding sites between miR-107 and SLC1A5 were predicted by starbase. **B**–**G** Th combination between miR-107 and SLC1A5 was verified using the dual-luciferase reporter assay (**B**, **C**), RIP assay (**D**, **E**) and RNA pull-down assay (**F**, **G**). **H** The mRNA expression of SLC1A5 was measured via qRT-PCR in normal and EC tissues. **I** The linear relationship between miR-107 and SLC1A5 was analyzed via Pearson’s correlation coefficient. **J**, **K** The SLC1A5 protein expression was detected via western blot in EC tissues (**J**) and cells (**K**). **L** The regulation of SLC1A5 protein level by miR-107 overexpression or inhibition was examined via western blot. ***P < 0.001, ****P < 0.0001

Starbase also predicted that the binding sites of miR-107 were presented in the 3’UTR sequence of SLC1A5 (Fig. 5A). Furthermore, the dual-luciferase reporter assay verified that co-transfection between SLC1A5 3’UTR-WT and miR-107 induced the luciferase signal inhibition in KYSE150 and TE1 cells (Fig. 5B, C). Also, the results from RIP assay (Fig. 5D, E) and pull-down assay (Fig. 5F, G) affirmed the interaction between miR-107 and SLC1A5. In EC tissue samples, SLC1A5 mRNA expression was markedly increased (Fig. 5H) and it was negatively correlated to miR-107 (*r* = -0.7776, *p* < 0.001) (Fig. 5I). Western blot analysis indicated that the protein level of SLC1A5 was also upregulated in EC tissues (Fig. 5J) and KYSE150/TE1 cells (Fig. 5K) in comparison to the normal control groups. The overexpression of miR-107 triggered the downregulation of SLC1A5 protein expression, while miR-107 inhibitor induced an opposite effect (Fig. 5L). All in all, miR-107 could target SLC1A5 to result in the direct expression inhibition of SLC1A5.

### SLC1A5 downregulation impeded the malignant behaviors of EC cells

**Fig. 6:**
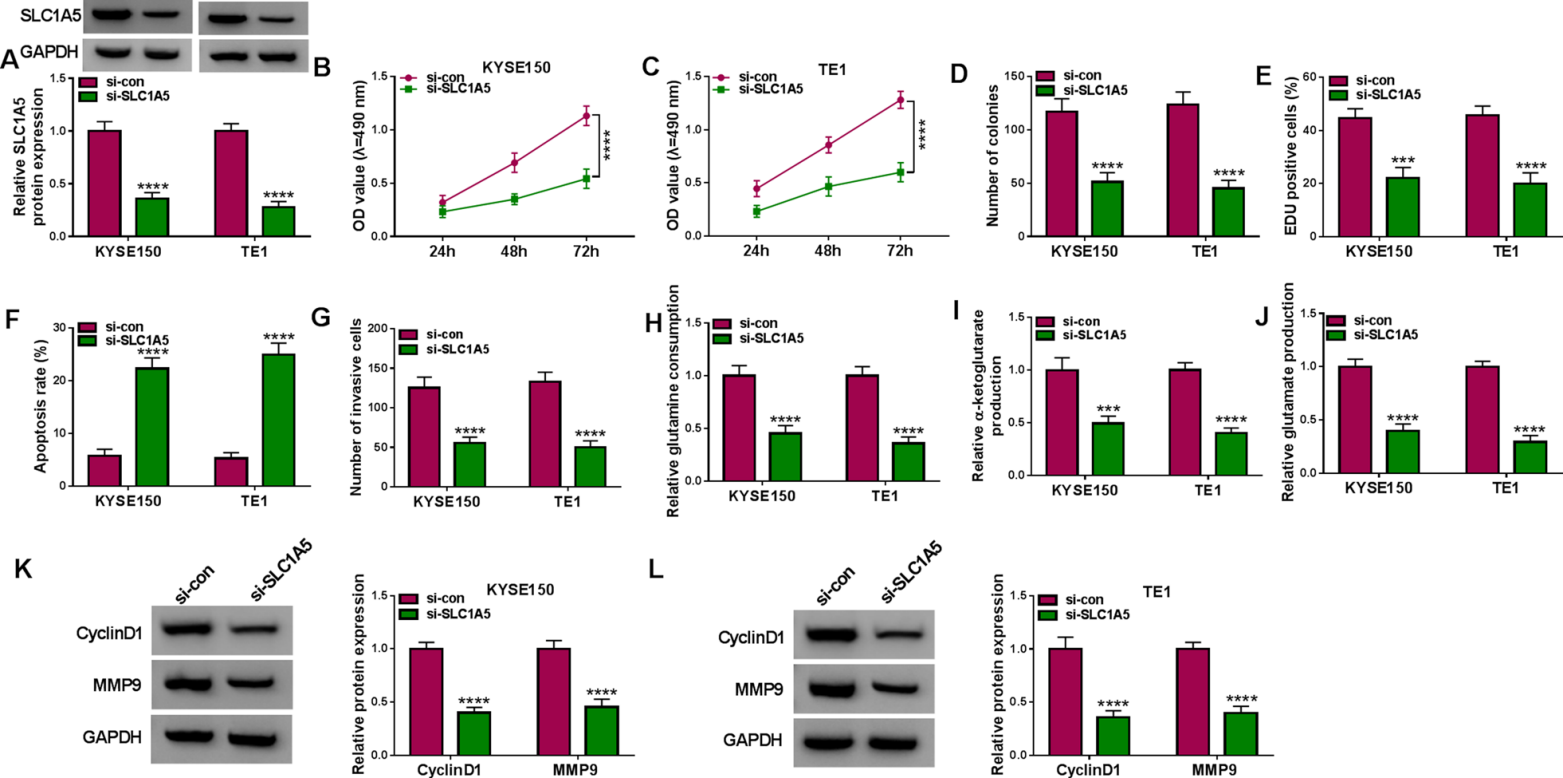
SLC1A5 downregulation impeded the malignant behaviors of EC cells. Transfection of si-con or si-SLC1A5 was performed in KYSE150 and TE1 cells. **A** The expression analysis of SLC1A5 was conducted by western blot. **B**–**E** Cell proliferation detection was conducted by MTT assay (**B, C**), colony formation assay (**D**) and EdU assay (**E**). **F, G** The evaluation of cell apoptosis (**F**) and invasion (**G**) was conducted by flow cytometry and transwell assay. **H**–**J** The measurement of glutamine metabolism was performed by the glutamine consumption (**H**), α-ketoglutarate production (**I**) and glutamate production (**J**) using the detection kits. **K**, **L** The protein quantification of CyclinD1 and MMP9 was performed by western blot. ***P < 0.001, ****P < 0.0001

The role of SLC1A5 in the progression of EC was investigated by the loss-function-method. The si-SLC1A5-mediated protein expression downregulation of SLC1A5 was conspicuous in KYSE150 and TE1 cells (Fig. 6A). The results of MTT assay (Fig. 6B, C), colony formation assay (Fig. 6D) and EdU assay (Fig. 6E) manifested that SLC1A5 knockdown led to the significant repression of cell proliferation. Cell apoptosis was promoted (Fig. 6F) but cell invasion was suppressed (Fig. 6G) by si-SLC1A5, contrasted with the si-con group. The glutamine consumption, α-ketoglutarate production and glutamate production were found to be reduced after the expression inhibition of SLC1A5 (Fig. 6H–J). Besides, CyclinD1 and MMP9 protein levels were lower in si-SLC1A5 group relative to si-con group (Fig. 6K, L). Taken together, the malignant progression of EC was blocked by downregulation of SLC1A5.

### MiR-107 severed as a tumor repressor in EC cells by targeting SLC1A5

**Fig. 7:**
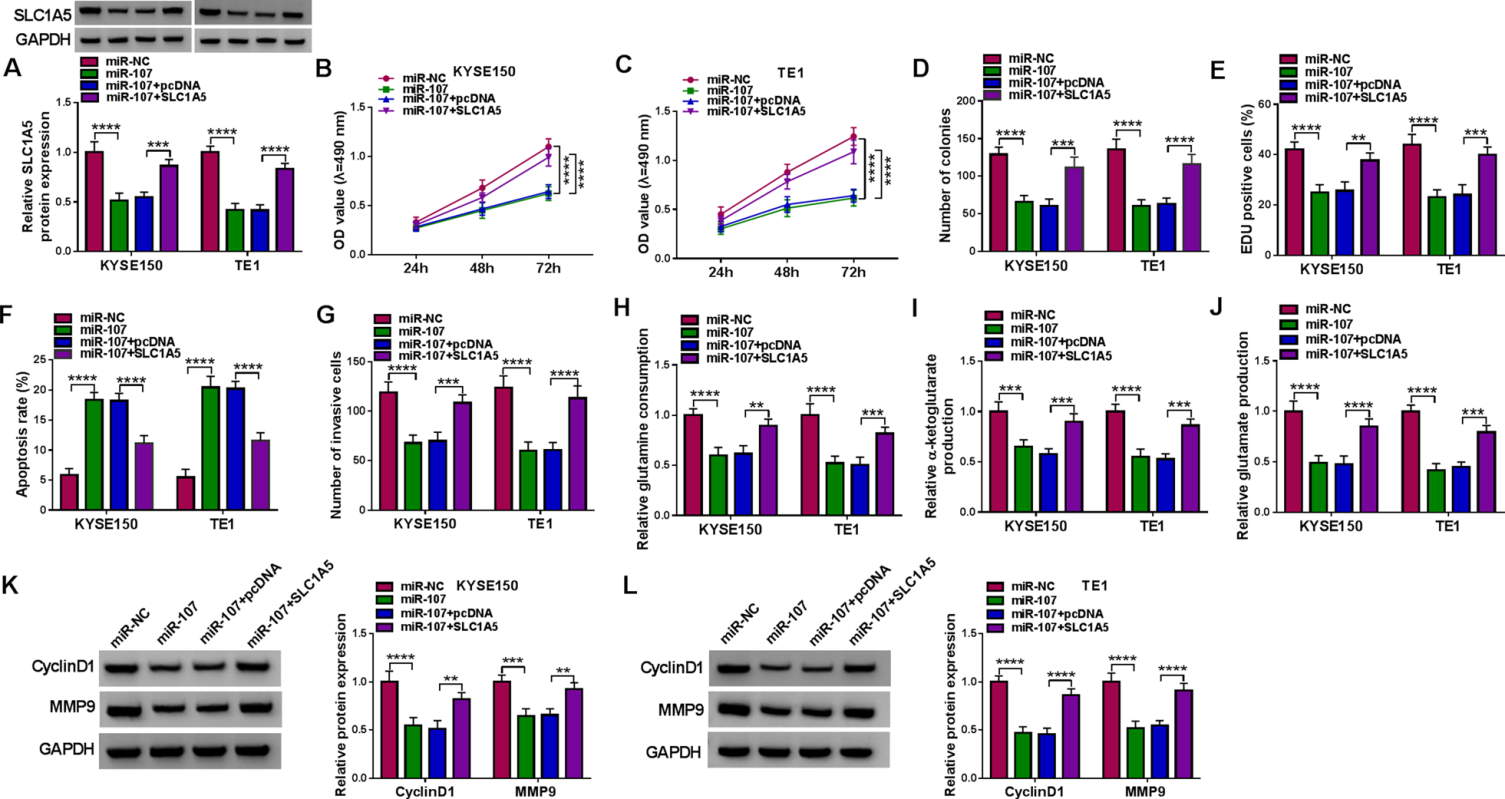
MiR-107 severed as a tumor repressor in EC cells by targeting SLC1A5. KYSE150 and TE1 cells were transfected with miR-NC, miR-107, miR-107 + pcDNA, miR-107 + SLC1A5. **A** Western blot was applied to assay the protein expression of SLC1A5. **B**–**E** MTT assay (**B**, **C**), colony formation assay (**D**) and EdU assay (**E**) were applied to analyze cell proliferation. **F**, **G** Flow cytometry and transwell assay were exploited to determine cell apoptosis (**F**) and invasion (**G**). **H–J** The glutamine consumption (**H**), α-ketoglutarate production (**I**) and glutamate production (**J**) by the detection kits were exploited to evaluate the glutamine metabolism. **K**, **L** Western blot was exploited to detect the protein levels of CyclinD1 and MMP9. **P < 0.01, ***P < 0.001, ****P < 0.0001

The miR-107/SLC1A5 axis was further researched in EC cells. The suppressive effect of miR-107 on the protein expression of SLC1A5 was returned by transfection of SLC1A5 (Fig. 7A). The overexpression of miR-107 was observed to inhibit cell proliferation (Fig. 7B–E) and facilitate cell apoptosis (Fig. 7F), whereas these influences were mitigated by the upregulation of SLC1A5. The repression of cell invasion (Fig. 7G) and the retardation of glutamine metabolism (Fig. 7H–J) caused by miR-107 were also partly abrogated by SLC1A5. Meanwhile, the introduction of SLC1A5 counteracted the miR-107-induced protein downregulation of CyclinD1 and MMP9 in kYSE150 and TE1 cells (Fig. 7K, L). In addition, overexpression of SLC1A5 has aggravated the anti-miR-107-induced promotion of cell proliferation, invasion and glutamine metabolism in KYSE150 and TE1 cells (Additional file 1: Fig. S1). The above findings implied that miR-107 played as a tumor inhibitor in EC cells by the direct downregulation of SLC1A5.

### Circ-SFMBT2 regulated the SLC1A5 expression by targeting miR-107

**Fig. 8:**
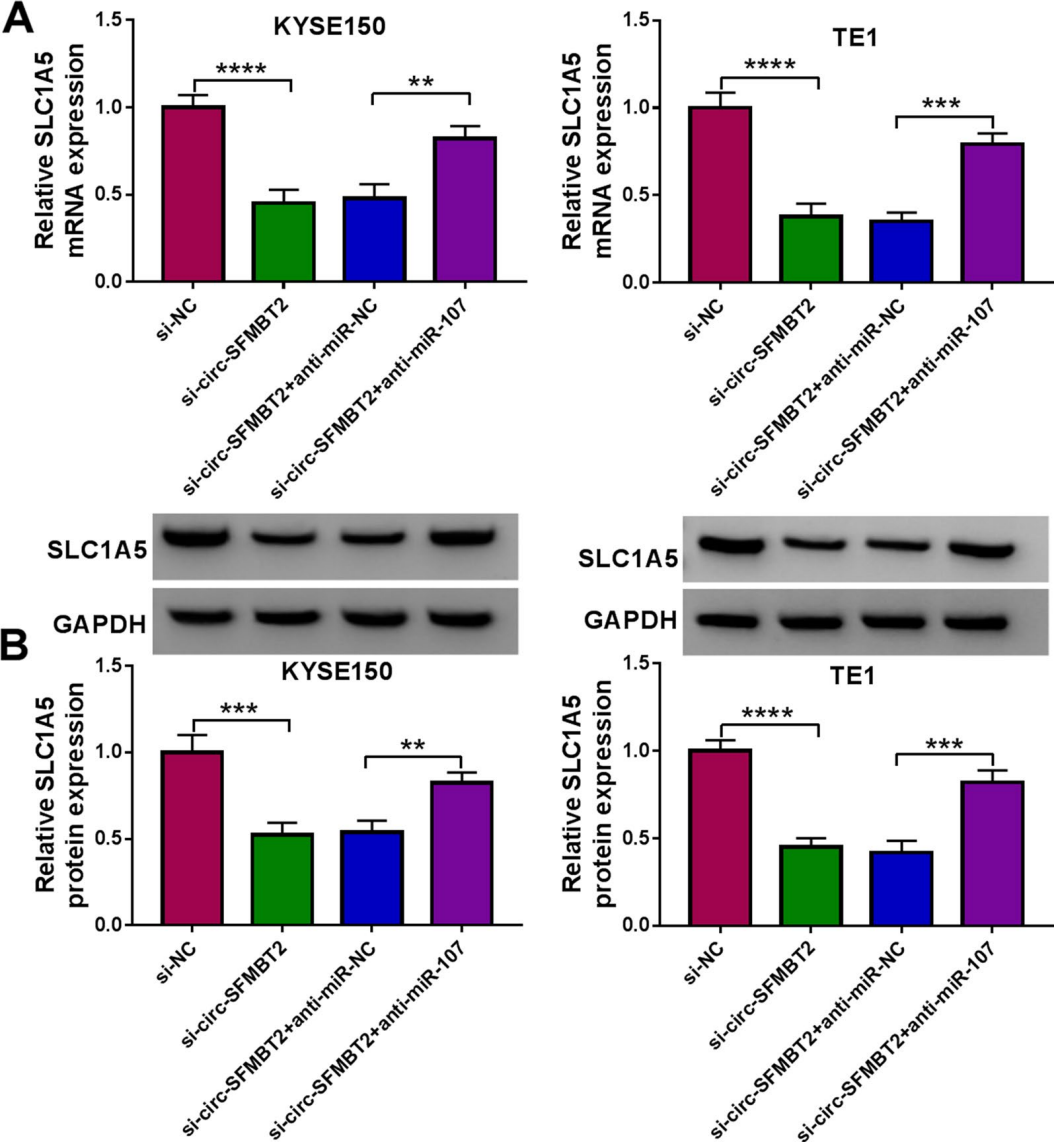
Circ-SFMBT2 regulated the SLC1A5 expression by targeting miR-107. **A**, **B** The mRNA (**A**) and protein (**B**) levels of SLC1A5 were measured by qRT-PCR and western blot after transfection of si-NC, si-circ-SFMBT2, si-circ-SFMBT2 + anti-miR-NC or si-circ-SFMBT2 + anti-miR-107. **P < 0.01, ***P < 0.001, ****P < 0.0001

Moreover, the regulatory effect of circ-SFMBT2 on the expression of SLC1A5 was studied in EC cells. The reverted transfection has revealed that anti-miR-107 eliminated the si-circ-SFMBT2-triggered downregulation of SLC1A5 mRNA and protein levels (Fig. 8A, B). The regulation of SLC1A5 by the circ-SFMBT2/miR-107 axis suggested the presence of circ-SFMBT2/miR-107/SLC1A5 network in EC cells.

### Knockdown of circ-SFMBT2 inhibited tumor progression of EC in vivo by regulating miR-107 and SLC1A5

**Fig. 9:**
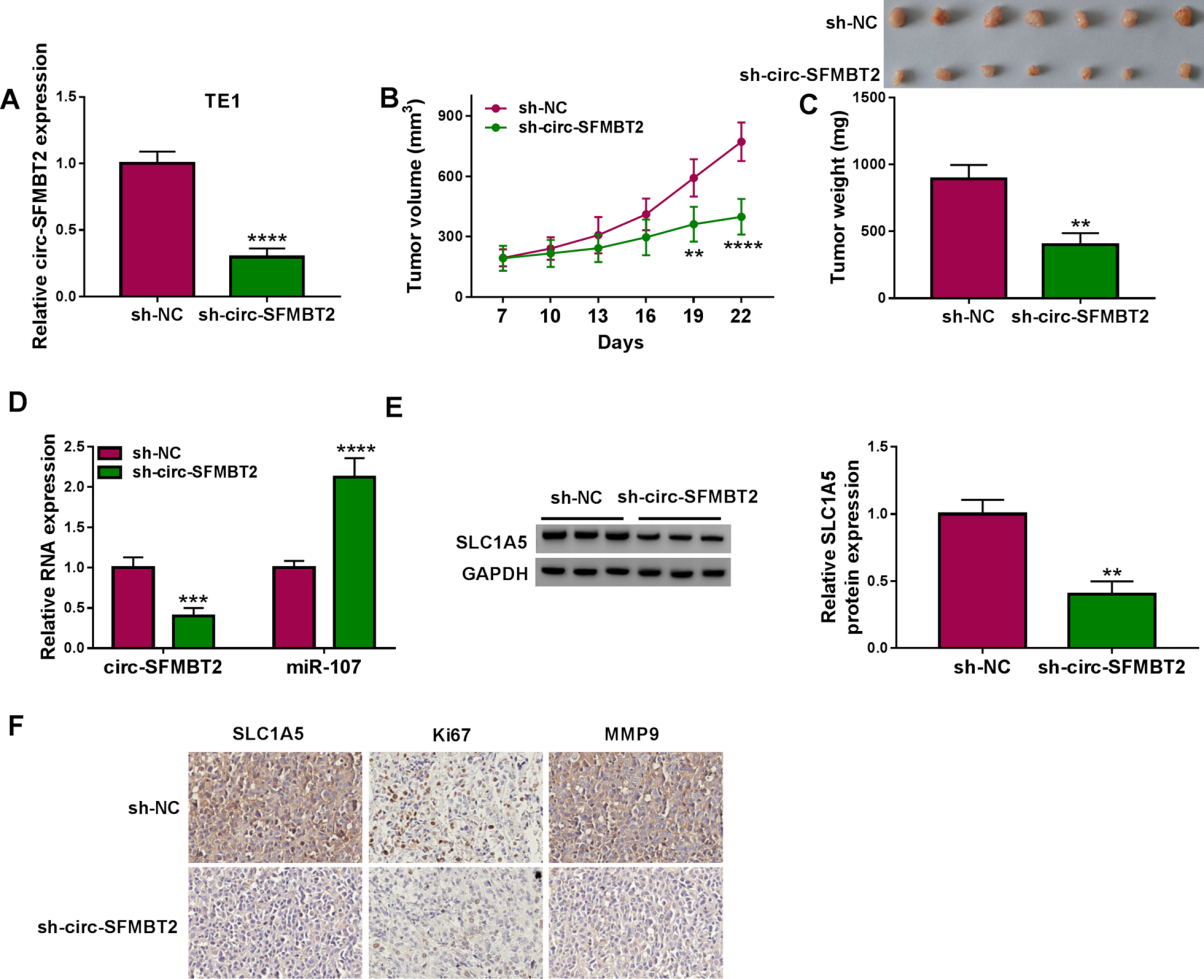
Knockdown of circ-SFMBT2 inhibited tumor progression of EC in vivo by regulating miR-107 and SLC1A5. **A** The circ-SFMBT2 expression was detected using qRT-PCR in TE1 cells of sh-NC or sh-circ-SFMBT2 group. **B**, **C** Tumor volume (**B**) and weight (**C**) were determined in sh-NC or sh-circ-SFMBT2 group. **D**, **E** The qRT-PCR and western blot were used to assay the expression of circ-SFMBT2 or miR-107 (**D**) and SLC1A5 (**E**) in tumor tissues. **F** IHC assay was performed for the protein analysis for SLC1A5, Ki67 and MMP9. **P < 0.01, ***P < 0.001, ****P < 0.0001

The stable TE1 cell line was constructed and the expression of circ-SFMBT2 was found to be decreased in sh-circ-SFMBT2 group compared with sh-NC group (Fig. 9A). All mice developed tumors. After mice were injected with cells for 22 days, tumor volume of sh-circ-SFMBT2 group was lower than that of sh-NC group (Fig. 9B). Tumor tissues were shown in Fig. 9C, and the tumor weight was also reduced by knockdown of circ-SFMBT2. The expression detection manifested the downregulation of circ-SFMBT2 and upregulation of miR-107 in tumor tissues of sh-circ-SFMBT2 group relative to sh-NC group (Fig. 9D). The circ-SFMBT2 inhibition incurred the protein repression of SLC1A5 in tumors (Fig. 9E). IHC assay demonstrated that the protein levels of SLC1A5, Ki67 and MMP9 in tumor tissues were suppressed by the silence of circ-SFMBT2, indicating that circ-SFMBT2 could promote tumor proliferation and metastasis in mice (Fig. 9F). I*n vivo* assay validated that circ-SFMBT2 regulated EC progression by the regulation of miR-107 and SLC1A5.

### Exosomes mediated the transfer of circ-SFMBT2 in EC cells

**Fig. 10:**
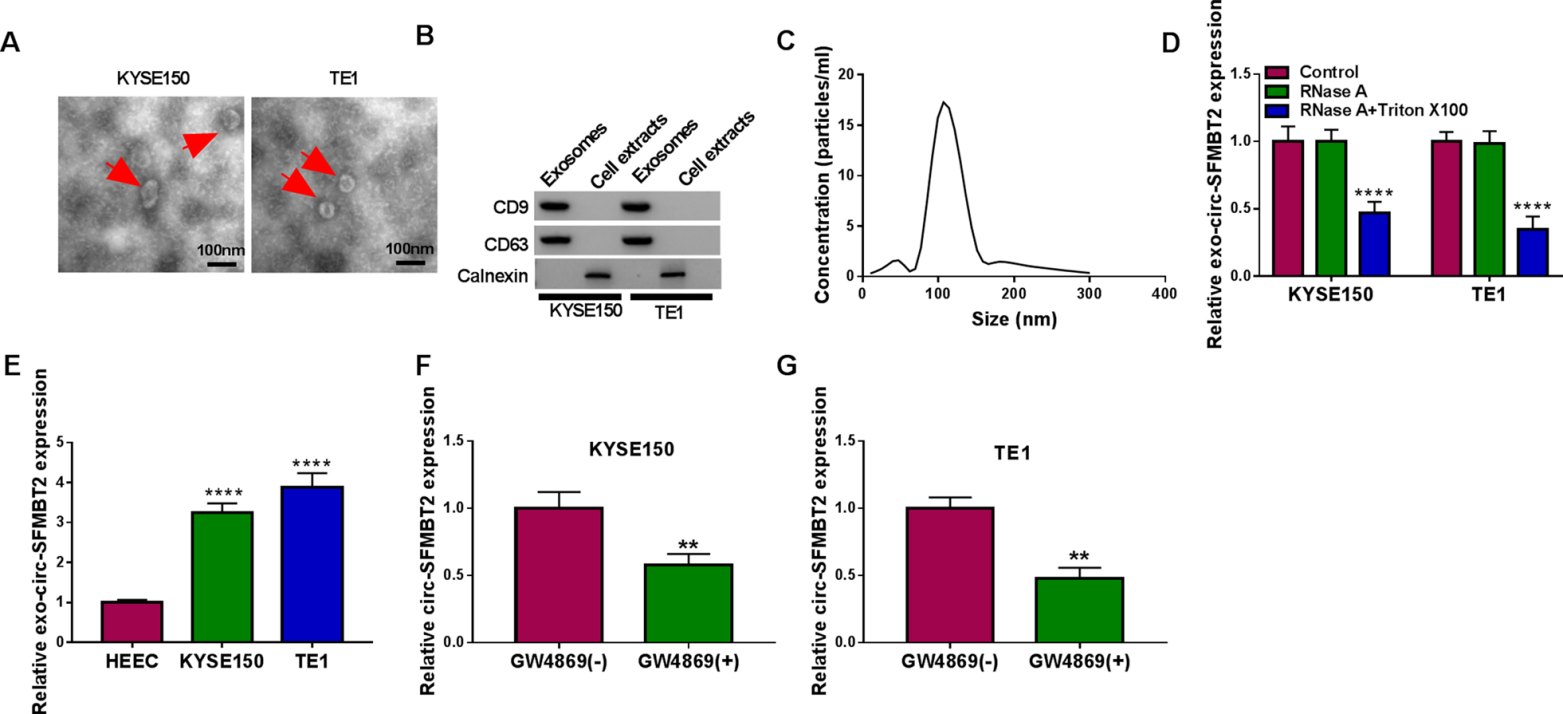
Exosomes mediated the transfer of circ-SFMBT2 in EC cells. **A** The morphological observation of exosome was performed under the TEM. **B** CD9, CD63 and Calnexin proteins were detected in exosomes by western blot. **C** The diameter detection of the isolated particles. **D** The qRT-PCR was applied for circ_0006174 expression analysis in control, RNase A, or RNase A + Triton X100 treatment group. **E** The circ-SFMBT2 expression in exosomes was examined using qRT-PCR. **F**, **G** The qRT-PCR was performed to determine the level of circ-SFMBT2 in KYSE150 and TE1 cells after the incubation of GW4869. **P < 0.01, ****P < 0.0001

The preliminary exploration of exosome in the function of circ-SFMBT2 was performed. Exosomes from KYSE150 and TE1 cells were exhibited as the bilayer molecules under the TEM (Fig. 10A). The exosomal makers CD9 and CD63 were detected in exosomes from cells but not cell extracts, while Calnexin (a protein associated with the endoplasmic reticulum and could be released by cells suffering mechanical damage) was not determined in exosomes (Fig. 10B). Thus, there were no cell debris and the pellets were identified as exosomes. The diameter detection indicated that the particles were about 100 nm, which was in consistent with the characteristic of exosome (Fig. 10C). The qRT-PCR data exhibited that RNase A did not affect the circ_0006174 level compared to control group, but circ-SFMBT2 expression was significantly downregulated by the co-treatment of RNase A and Triton X100 (Fig. 10D). Thus, circ-SFMBT2 was mainly packaged in the membranes. The qRT-PCR showed that the circ-SFMBT2 expression was upregulated in exosomes from KYSE150 and TE1 cells compared with HEEC cells (Fig. 10E). Moreover, the level of circ-SFMBT2 was downregulated after the incubation of exosomal inhibitor GW4869 (Fig. 10F, G). Altogether, exosomes might be involved in the transfer the circ-SFMBT2.

## Discussion

In this study, we firstly reported the oncogenic properties of circ-SFMBT2 in EC by performing experiments *in vitro* and *in vivo*. More importantly, our results indicated the regulation of circ-SFMBT2/miR-107/SLC1A5 axis in EC progression.

The expression detection for circ-SFMBT2 revealed that it was highly expressed in EC tissue samples and cell lines. The dysregulated circRNAs have been implicated in the regulation of cancer development. For instance, knockdown of circRNA_0007534 suppressed proliferation and accelerated apoptosis in cervical cancer cells [21]. CircGFRA1 promoted the tumor growth and metastasis of hepatocellular carcinoma [22], and circNDUFB2 repressed the malignant progression of non-small cell lung cancer [23]. MTT assay, colony formation assay and EdU assay were used to assess the effect of circ-SFMBT2 on cell proliferation. The downregulation of circ-SFMBT2 suppressed the proliferation ability of EC cells. Meanwhile, cell apoptosis was enhanced and cell invasion was blocked by the silence of circ-SFMBT2. These results suggested the pro-cancerous effect of circ-SFMBT2 on the EC progression.

Glutaminolysis is a metabolic route essential for cancer cell survival and growth [24]. A handful of circRNAs have been found to regulate the process of glutaminolysis in different cancers. Yue *et al*. showed that circ_0004104 contributed to the glutaminolysis of gastric cancer cells [25], and Zhen *et al*. declared that circHMGCS1 could facilitate the glutamine metabolism in hepatoblastoma cells [26]. Herein, we found that the inhibition of circ-SFMBT2 reduced the consumption of glutamine and the synthesis of glutamate or α-ketoglutarate. Thus, silencing circ-SFMBT2 retarded the glutamine metabolism in EC progression.

CircLPAR3 has been exhibited to expedite migration and invasion of EC cells by sponging miR-198 [27]. The promoting effects of circRAD23B on EC cell proliferation and invasion were also achieved by sequestering miR-5095 [28]. Our target analysis suggested that miR-107 interacted with circ-SFMBT2 and the reverted experiments validated that the functional regulation of circ-SFMBT2 was associated with the regulation of miR-107 in EC cells. In addition, miR-107 could target SLC1A5 and it blocked the EC progression by the direct downregulation of SLC1A5 expression. SLC1A5 is a mitochondrial glutamine transporter in cancer metabolic reprogramming [29]. The functional assays for SLC1A5 manifested that it could accelerate cell proliferation, invasion and glutaminolysis of EC cells. Due to the regulatory effect of circ-SFMBT2 on the SLC1A5 level by targeting miR-107, the oncogenic function of circ-SFMBT2 in EC development was considered to be related to the miR-107-mediated SLC1A5 expression change. The effect of circ-SFMBT2 on tumorigenesis *in vivo* was also attributed to the regulation of miR-107 and SLC1A5 levels.

Extracellular exosomes can transport different regulatory molecules from donor cells to acceptant cells, and they have important roles in cancer diagnosis and treatment [30]. Exosomal hsa_circ_0051443 has been reported as a predictive and therapeutic target for hepatocellular carcinoma [31]. Tumor-released exosomal circ-PDE8A enhanced cell invasion in pancreatic cancer, and exosomal circ-PDE8A was a potential marker for cancer diagnosis or progression [32]. We have performed a preliminary exploration for exosomes and circ-SFMBT2 in EC cells. High expression of circ-SFMBT2 was found in exosomes from EC cells, and the exosomal inhibitor could downregulate the circ-SFMBT2 level in EC cells. Exosomes might transmit circ-SFMBT2 to aggravate the malignant progression of EC. The association of exosome and circ-SFMBT2/miR-107/SLC1A5 axis remains to be researched in the future study.

## Conclusion

To summarize, circ-SFMBT2 was affirmed as a tumorigenic molecule to promote EC progression and glutamine metabolism via targeting the miR-107/SLC1A5 axis. This study indicated that circ-SFMBT2 could be a useful diagnostic and therapeutic biomarker for EC.

## Supplementary Information

**Additional file 1: Fig. S1**. Overexpression of SLC1A5 enhanced the anti-miR-107-mediated tumor progression in EC cells. KYSE150 and TE1 cells were transfected with anti-miR-NC, anti-miR-107, anti-miR-107+pcDNA, anti-miR-107+SLC1A5. (A) SLC1A5 protein detection was performed using western blot. (B-E) The proliferation examination was performed using MTT assay (B-C), colony formation assay (D) and EdU assay (E). (F) Cell invasion ability was analyzed using transwell assay. (G-I) The glutamine metabolism was assessed through glutamine consumption (G), α-ketoglutarate production (H) and glutamate production (I) by the corresponding kits. *P < 0.05, **P < 0.01, ***P < 0.001, ****P < 0.0001.

## Data Availability

Not applicable.

## Abbreviations

circ-SFMBT2: CircRNA Scm-like with four malignant brain tumor domains 2
EC: Esophageal cancer
SLC1A5: Solute-linked carrier family A1 member 5
HEEC: Human esophageal epithelial cell line

## References

[1] Liu K, Zhao T, Wang J, Chen Y, Zhang R, Lan X, Que J (2019). Etiology, cancer stem cells and potential diagnostic biomarkers for esophageal cancer. Cancer Lett.

[2] Bollschweiler E, Plum P, Monig SP, Holscher AH (2017). Current and future treatment options for esophageal cancer in the elderly. Expert Opin Pharmacother.

[3] Huang FL, Yu SJ (2018). Esophageal cancer: risk factors, genetic association, and treatment. Asian J Surg.

[4] Torsin LI, Petrescu GED, Sabo AA, Chen B, Brehar FM, Dragomir MP, Calin GA (2021). Editing and Chemical modifications on non-coding rnas in cancer: a new tale with clinical significance. Int J Mol Sci.

[5] Goodall GJ, Wickramasinghe VO (2021). RNA in cancer. Nat Rev Cancer.

[6] Kristensen LS, Andersen MS, Stagsted LVW, Ebbesen KK, Hansen TB, Kjems J (2019). The biogenesis, biology and characterization of circular RNAs. Nat Rev Genet.

[7] Xu T, Wang M, Jiang L, Ma L, Wan L, Chen Q, Wei C, Wang Z (2020). CircRNAs in anticancer drug resistance: recent advances and future potential. Mol Cancer.

[8] Wang T, Wang J, Ren W, Chen S, Cheng YF, Zhang XM (2020). CircRNA-0008717 promotes cell proliferation, migration, and invasion by regulating miR-203/Slug in esophageal cancer cells. Ann Transl Med.

[9] Chen Z, Yao N, Gu H, Song Y, Ye Z, Li L, Lu P, Shao Q (2020). Circular RNA_LARP4 sponges miR-1323 and hampers progression of esophageal squamous cell carcinoma through modulating PTEN/PI3K/AKT pathway. Dig Dis Sci.

[10] Sun H, Xi P, Sun Z, Wang Q, Zhu B, Zhou J, Jin H, Zheng W, Tang W, Cao H, Cao X (2018). Circ-SFMBT2 promotes the proliferation of gastric cancer cells through sponging miR-182–5p to enhance CREB1 expression. Cancer Manag Res..

[11] Feng D, Xu Y, Hu J, Zhang S, Li M, Xu L (2020). A novel circular RNA, hsa-circ-0000211, promotes lung adenocarcinoma migration and invasion through sponging of hsa-miR-622 and modulating HIF1-alpha expression. Biochem Biophys Res Commun.

[12] Wang L, Li K, Wang C, Shi X, Yang H (2019). miR-107 regulates growth and metastasis of gastric cancer cells via activation of the PI3K-AKT signaling pathway by down-regulating FAT4. Cancer Med.

[13] Fu Y, Lin L, Xia L (2019). MiR-107 function as a tumor suppressor gene in colorectal cancer by targeting transferrin receptor 1. Cell Mol Biol Lett.

[14] Sharma P, Saini N, Sharma R (2017). miR-107 functions as a tumor suppressor in human esophageal squamous cell carcinoma and targets Cdc42. Oncol Rep.

[15] Lin J, Yang T, Peng Z, Xiao H, Jiang N, Zhang L, Ca D, Wu P, Pan Q (2018). SLC1A5 silencing inhibits esophageal cancer growth via cell cycle arrest and apoptosis. Cell Physiol Biochem.

[16] Tang Q, Li M, Chen L, Bi F, Xia H (2018). miR-200b/c targets the expression of RhoE and inhibits the proliferation and invasion of non-small cell lung cancer cells. Int J Oncol.

[17] Zhong Y, Wang Y, Zhang C, Hu Y, Sun C, Liao J, Wang G (2019). Identification of long non-coding RNA and circular RNA in mice after intra-tracheal instillation with fine particulate matter. Chemosphere.

[18] Livak KJ, Schmittgen TD (2001). Analysis of relative gene expression data using real-time quantitative PCR and the 2(-Delta Delta C(T)) Method. Methods.

[19] Li M, Luan L, Liu Q, Liu Y, Lan X, Li Z, Liu W (2019). MiRNA-199a-5p protects against cerebral ischemic injury by down-regulating DDR1 in rats. World Neurosurg..

[20] Vanhove K, Derveaux E, Graulus GJ, Mesotten L, Thomeer M, Noben JP, Guedens W, Adriaensens P (2019). Glutamine addiction and therapeutic strategies in lung cancer. Int J Mol Sci.

[21] Sun Q, Qi X, Zhang W, Li X (2021). Knockdown of circRNA_0007534 suppresses the tumorigenesis of cervical cancer via miR-206/GREM1 axis. Cancer Cell Int.

[22] Lv S, Li Y, Ning H, Zhang M, Jia Q, Wang X (2021). CircRNA GFRA1 promotes hepatocellular carcinoma progression by modulating the miR-498/NAP1L3 axis. Sci Rep.

[23] Li B, Zhu L, Lu C, Wang C, Wang H, Jin H, Ma X, Cheng Z, Yu C, Wang S, Zuo Q, Zhou Y, Wang J, Yang C, Lv Y, Jiang L, Qin W (2021). circNDUFB2 inhibits non-small cell lung cancer progression via destabilizing IGF2BPs and activating anti-tumor immunity. Nat Commun.

[24] Cardoso HJ, Figueira MI, Vaz CV, Carvalho TMA, Bras LA, Madureira PA, Oliveira PJ, Sardao VA, Socorro S (2021). Glutaminolysis is a metabolic route essential for survival and growth of prostate cancer cells and a target of 5alpha-dihydrotestosterone regulation. Cell Oncol (Dordr).

[25] Yue F, Peng K, Zhang L, Zhang J. Circ_0004104 Accelerates the progression of gastric cancer by regulating the miR-539–3p/RNF2 axis. Dig Dis Sci. 2021:1-12 10.1007/s10620-020-06802-5 33449226

[26] Zhen N, Gu S, Ma J, Zhu J, Yin M, Xu M, Wang J, Huang N, Cui Z, Bian Z, Sun F, Pan Q (2019). CircHMGCS1 promotes hepatoblastoma cell proliferation by regulating the IGF signaling pathway and glutaminolysis. Theranostics.

[27] Shi Y, Fang N, Li Y, Guo Z, Jiang W, He Y, Ma Z, Chen Y (2020). Circular RNA LPAR3 sponges microRNA-198 to facilitate esophageal cancer migration, invasion, and metastasis. Cancer Sci.

[28] Lan X, Liu X, Sun J, Yuan Q, Li J (2019). CircRAD23B facilitates proliferation and invasion of esophageal cancer cells by sponging miR-5095. Biochem Biophys Res Commun.

[29] Yoo HC, Park SJ, Nam M, Kang J, Kim K, Yeo JH, Kim JK, Heo Y, Lee HS, Lee MY, Lee CW, Kang JS, Kim YH, Lee J, Choi J, Hwang GS, Bang S, Han JM (2020). A variant of SLC1A5 Is a mitochondrial glutamine transporter for metabolic reprogramming in cancer cells. Cell Metab.

[30] Shehzad A, Islam SU, Shahzad R, Khan S, Lee YS (2021). Extracellular vesicles in cancer diagnostics and therapeutics. Pharmacol Ther.

[31] Chen W, Quan Y, Fan S, Wang H, Liang J, Huang L, Chen L, Liu Q, He P, Ye Y (2020). Exosome-transmitted circular RNA hsa_circ_0051443 suppresses hepatocellular carcinoma progression. Cancer Lett.

[32] Li Z, Yanfang W, Li J, Jiang P, Peng T, Chen K, Zhao X, Zhang Y, Zhen P, Zhu J, Li X (2018). Tumor-released exosomal circular RNA PDE8A promotes invasive growth via the miR-338/MACC1/MET pathway in pancreatic cancer. Cancer Lett.

