# Supplementary figures and images for: Circ-SFMBT2 drives the malignant phenotypes of esophageal cancer by the miR-107-dependent regulation of SLC1A5
Source: Cancer Cell Int. 2021 Sep 16;21:495. doi: 10.1186/s12935-021-02156-8 (PMC8447765; doi:10.1186/s12935-021-02156-8)

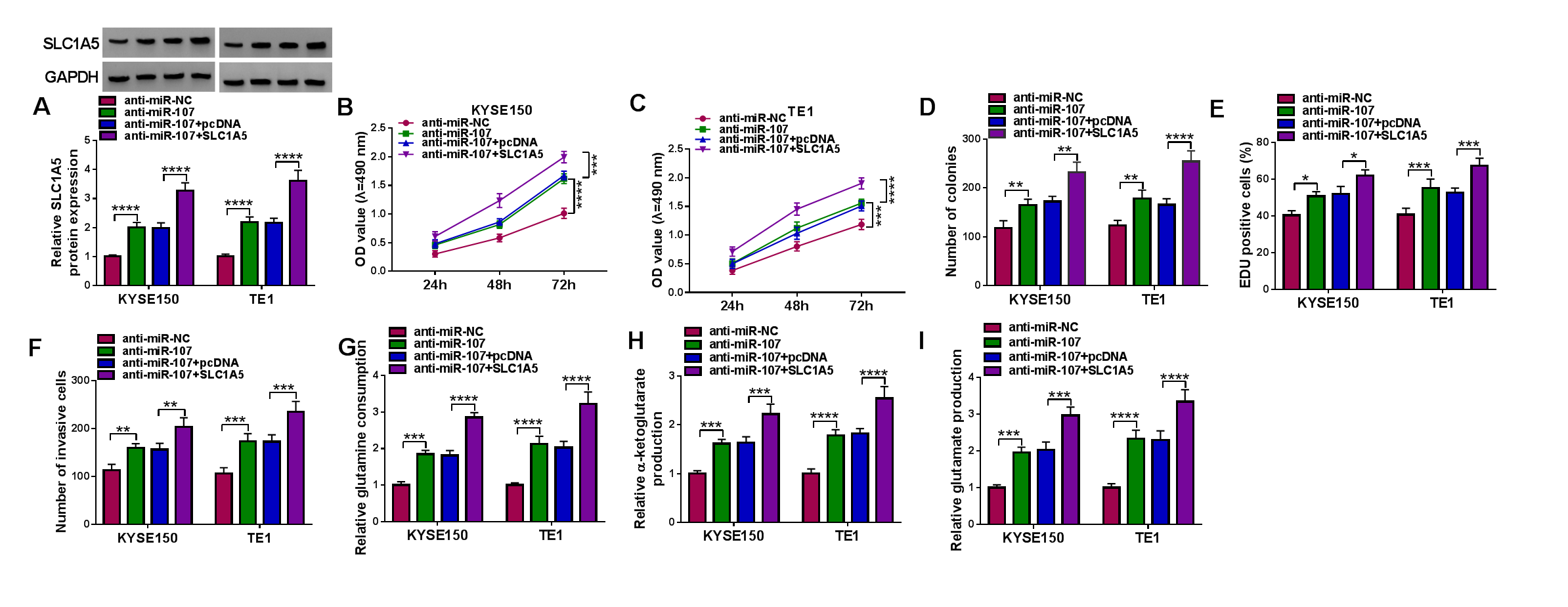

Supplement: Supplementary file 1 — Additional file 1: Fig. S1. Overexpression of SLC1A5 enhanced the anti-miR-107-mediated tumor progression in EC cells. KYSE150 and TE1 cells were transfected with anti-miR-NC, anti-miR-107, anti-miR-107+pcDNA, anti-miR-107+SLC1A5. (A) SLC1A5 protein detection was performed using western blot. (B-E) The proliferation examination was performed using MTT assay (B-C), colony formation assay (D) and EdU assay (E). (F) Cell invasion ability was analyzed using transwell assay. (G-I) The glutamine metabolism was assessed through glutamine consumption (G), α-ketoglutarate production (H) and glutamate production (I) by the corresponding kits. *P < 0.05, **P < 0.01, ***P < 0.001, ****P < 0.0001. [file 12935_2021_2156_MOESM1_ESM.tif]
